# A Tightly Coupled Multibody Dynamics and Multi-Sensor Fusion Algorithm for Simultaneous Kinematics and Kinetics Estimation

**DOI:** 10.3390/s26123697

**Published:** 2026-06-10

**Authors:** Hassan Osman, Daan de Kanter, Jelle Boelens, Manon Kok, Ajay Seth

**Affiliations:** 1 Department of Biomechanical Engineering, Delft University of Technology, 2628 CD Delft, The Netherlands; daandekanter1996@gmail.com (D.d.K.); a.seth@tudelft.nl (A.S.); 2 Delft Center for Systems and Control, Delft University of Technology, 2628 CD Delft, The Netherlands; m.kok-1@tudelft.nl

**Keywords:** sensor fusion, motion capture, iterated extended Kalman filter (IEKF), kinematics, kinetics, IMU, inertial sensors, movement tracking

## Abstract

Inertial Measurement Units (IMUs) enable portable, multibody motion capture in diverse environments beyond the laboratory, making them a desirable choice for diagnosing mobility disorders and supporting rehabilitation in clinical or home settings. However, challenges associated with IMU measurements, including magnetic distortions and errors due to integration drift, complicate their broader use for motion capture. In this work, we propose a tightly coupled motion-capture approach that directly integrates IMU measurements with multibody dynamic models via an iterated extended Kalman filter to simultaneously estimate the system’s kinematics and kinetics. By enforcing the complete multibody system dynamics and utilizing only accelerometer and gyroscope data, our method accurately estimates joint kinematics and kinetics. Our algorithm is designed to fuse different sensor data, such as optical motion-capture measurements and joint torque readings, to further enhance estimation accuracy. We validated our approach using highly accurate ground-truth data from a 3-degree-of-freedom pendulum and a 6-degree-of-freedom collaborative robot. We demonstrate a maximum root-mean-square difference of 3.75° in the pendulum’s computed joint angles with respect to the marker motion-capture inverse kinematics. For the robot, we observed a maximum joint angle root-mean-square difference of 3.24° with respect to the joint encoders, while the maximum joint angle root-mean-square difference of the optical motion-capture inverse kinematics with respect to the encoders was 1.16°. With regard to kinetic estimates, we report a maximum joint torque root-mean-square difference of 3.02 Nm in the pendulum with respect to the marker motion-capture inverse dynamics and 4.27 Nm in the robot relative to its joint torque sensors.

## 1. Introduction

Motion-capture systems are used in a wide range of applications, such as sports, film animation, video games and, importantly, the diagnosis and treatment of human movement disorders. They provide a method to analyze multibody movements [1,2,3,4]. Analysis of human movement begins with motion-capture systems [5] that provide measurements from a collection of sensors, from which the subject’s kinematics and kinetics can be evaluated. Motion-capture measurements typically take the form of 3D marker positions, 3D sensor orientations from Inertial Measurement Units (IMUs), or joint positions from markerless video-based systems [6,7].

IMUs allow multibody motion capture in more varied environments and settings compared to optical marker-based systems, which are typically restricted to controlled laboratory conditions [6,8]. This flexibility makes IMU-based systems particularly valuable for applications such as patient rehabilitation, where portability, accessibility and privacy are important [3]. However, due to limitations in accuracy, interpretability, and sensor-to-body calibration, IMU-based systems have not been widely adopted in clinical applications [9,10]. In this work, we focus on an estimation framework based on a tightly coupled motion-capture algorithm that leverages knowledge of the multibody system’s dynamics, which are directly related to the IMU’s accelerometer and gyroscope measurements, to simultaneously estimate system kinematics and kinetics, expressed in biomechanical motion axes [11]. By enforcing dynamic consistency between the estimated kinematics and kinetics, our approach aims to improve the accuracy of IMU-based motion estimation.

IMUs capture linear acceleration and angular velocity using accelerometers and gyroscopes [12]. In addition, IMUs typically incorporate a magnetometer to measure the local magnetic field intensity. Using these measurements, the orientation of the IMU can be estimated [12]. Inaccuracies in IMU-derived orientations arise from multiple sources: magnetic distortions (especially indoors), gyroscope bias and noise, and the difficulty of separating inertial acceleration from gravitational acceleration during motion. Consequently, orientation estimates contain errors that propagate to subsequent joint angle estimates. While these angular estimates are often used directly for kinematic analysis, in cases where kinetics are also required, they are typically low-pass-filtered and then differentiated twice and filtered to compute the joint torques using inverse dynamics (ID) [13]. Due to this process, the resulting kinetics are often inconsistent with the kinematic constraints imposed by joint definitions and with external forces measured by additional sensors such as force-plates [12,13,14,15,16,17,18].

Musculoskeletal modeling software such as OpenSim [19,20,21] enables joint angle estimation while imposing joint mobility constraints through inverse kinematics (IK). OpenSense [22], an open-source algorithm within OpenSim (version 4.4), utilizes IMU orientations in conjunction with OpenSim’s internal coordinate formulation [23] to estimate multibody joint kinematics via IK. In this method, virtual IMUs are placed on the model’s body segments with the same initial orientations as the real IMUs. In each time frame of movement, IK is solved for the model’s joint angles that best reproduce the experimentally measured IMU orientations by minimizing the angular distance (error) between the orientations of the virtual and experimental IMUs. Because the measured IMU orientations are directly used to estimate joint angles, errors in IMU orientation estimates propagate to the resulting kinematics, making accurate IMU orientation estimation critical for this approach. Alternatively, kinematically coupled approaches directly integrate joint kinematic definitions with raw IMU measurements, bypassing the need for precomputed orientations. For example, Weygers et al. [24] enforce joint kinematic constraints by requiring consistency in acceleration at the joint center shared by adjacent body segments, as measured by the IMUs mounted on each segment. However, while this approach improves kinematic consistency, joint kinetics are not estimated directly.

Incorporating the multibody dynamics of the system, along with appropriate joint definitions, has been found to improve the accuracy of the estimated kinematics and kinetics [25]. Similar to the works of Dorschky et al. [25], Haraguchi and Hase [26], Nitschke et al. [27], and Koelewijn et al. [28], we leverage dynamic models to estimate joint kinematics and kinetics directly from IMU measurements (linear accelerations and angular velocities). However, our approach differs in several key aspects. First, unlike Dorschky et al. [25], who employed a planar model, we use the full multibody system dynamics to enforce the system equations of motion as part of the estimation. Second, contrary to Nitschke et al. [27], we validate our method using real IMU measurements rather than relying on simulated inertial data generated from marker-based motion capture. Third, unlike trajectory optimization approaches such as that of Haraguchi and Hase [26], which require predefined movement parameterizations (e.g., fixed nodes per gait cycle) and are limited to periodic movements with known cycle boundaries, our method performs state estimation without requiring prior knowledge of movement type, duration, or periodicity. Similarly, Koelewijn et al. [28] require a predefined time discretization over a fixed movement window and estimate the entire trajectory simultaneously, whereas our approach is sequential and makes no such assumptions. We present an iterated extended Kalman filter (IEKF)-based algorithm that enables the use of different sensor modalities and measurement types. We demonstrate this extensibility by integrating marker-based motion capture and zero-torque updates (described in detail in Section 2) along with IMU measurements. We tested our algorithm using real sensor measurements on two experimental setups: a 6-DoF KUKA robot (KUKA AG, Augsburg, Germany) and a 3-DoF pendulum, each of which provided accurate ground-truth data.

## 2. Methodology

**Figure 1:**
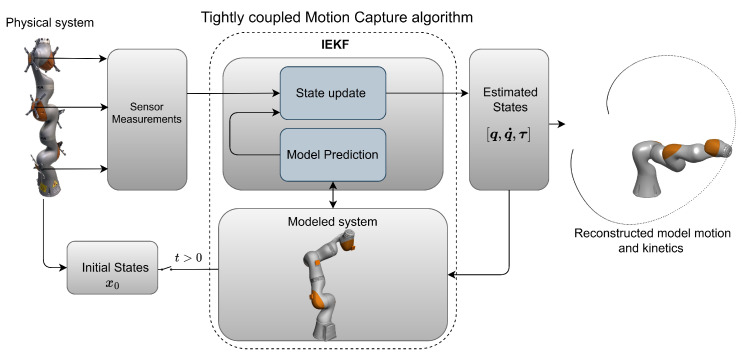
Overview of the tightly coupled motion-capture algorithm (TCMA).

To exploit the underlying multibody dynamics in the movement estimation problem, we developed an algorithm to directly couple the multibody system’s equations of motion with IMU sensor measurements (Figure 1). Estimating the system’s kinematic and kinetic states directly from the system dynamics requires formulating a continuous nonlinear state-space model that describes the measurements in terms of the system states [12,29,30,31]. In this work, we leverage OpenSim (version 4.4) [20], an open-source software for musculoskeletal modeling and simulation, to evaluate the equations of motion and kinematic Jacobians of the multibody system, and we use it to derive different sensor measurement models [32].

The motion of a multibody system with $N_{J}$ joints, $N_{B}$ body segments and $N_{D}$ joint angles at each time instant *t* is estimated in terms of joint angles $q={\left(\begin{array}{c} q_{1},q_{2},\ldots ,q_{N_{D}} \end{array}\right)}^{T}$, joint angular speeds $\dot{q}={\left(\begin{array}{c} {\dot{q}}_{1},{\dot{q}}_{2},\ldots ,{\dot{q}}_{N_{D}} \end{array}\right)}^{T}$ and joint torques $\tau ={\left(\begin{array}{c} \tau_{1},\tau_{2},\ldots ,\tau_{N_{D}} \end{array}\right)}^{T}$. Hence, the state vector x that we are interested in estimating is given by

(1) $$x^{T}=\left(\begin{array}{ccc} q^{T} & {\dot{q}}^{T} & \tau^{T} \end{array}\right),$$

where $q\in R^{N_{D}}$, $\dot{q}\in R^{N_{D}}$ and $\tau \in R^{N_{D}}$.

### 2.1. General Multibody State-Space Model

The multibody system dynamics describes the dynamics of the states in (1) as

(2) $$\tau =\bar{M}\left(q\right)\ddot{q}+\underset{\tau^{I}\left(q,\dot{q}\right)}{\underset{︸}{C\left(q,\dot{q}\right)+G\left(q\right)}},$$

for which $\bar{M}\left(q\right)$ is a symmetric, positive definite mass matrix, $C(q,\dot{q})$ is a function that groups all the velocity terms, and G(q) is a function that represents the gravity force component [19,33]. Furthermore, $\tau^{I}\left(q,\dot{q}\right)$ is a function representing the torque that results from gravity, the Coriolis force and the centrifugal force. Using (2), the joint angular accelerations can be expressed as

(3) $$\ddot{q}=\bar{M}{\left(q\right)}^{-1}\left(\tau -\tau^{I}\left(q,\dot{q}\right)\right).$$

The system’s dynamic model is expressed to include process noise, which accounts for uncertainties and disturbances in the system as follows:

(4) $$\dot{x}=g\left(x\right)+e_{p}.$$

where $e_{p}$ is zero-mean Gaussian noise with covariance $Q\in R^{3N_{D}\times 3N_{D}}$. The joint torques are assumed to evolve in a random-walk process [34] with $\dot{\tau }=e_{p_{\tau }}$, where $e_{p_{\tau }}∼N\left(0,Q_{\tau }\right)$. Since the process noise is assumed to only affect the torque states, the process noise covariance matrix Q has a block-diagonal structure with zero blocks for the generalized coordinates and velocities and $Q_{\tau }$ for the joint torques.

The system’s measurement model relates the states to the measurements as follows:

(5) $$y=h\left(x\right)+e_{\mathrm{meas}}.$$

Here, $h:R^{3N_{D}}\to R^{N_{y}}$ is a function that maps the states to the $N_{y}$ sensor measurements, and $e_{\mathrm{meas}}∼N\left(0,R\right)$ is the measurement error, where $R=\mathrm{blkdiag}(\Sigma_{\omega ,s_{1}},\Sigma_{\dot{v},s_{1}},\Sigma_{p,m_{1}},\Sigma_{\tau ,\phi_{1}},\ldots )$. These measurements consist of IMU data, optical markers, and zero-torque updates, which will be described in Section 2.2 and Section 2.3.

### 2.2. IMU Sensor Measurement Model

We assume that each body segment has at least one IMU attached. The total number of IMUs on the system’s body segments is $N_{I}$. Each IMU *s*, with $s=1,\ldots ,N_{I}$, has a gyroscope that measures the angular velocity $\omega_{s}^{s}$ and an accelerometer that measures linear acceleration ${\dot{v}}_{s}^{s}$, where the subscript indicates the measurement of sensor *s*, and the superscript denotes the frame in which the measurement is expressed. This notation will be used throughout the rest of this paper. The axes of the frame *s* are assumed to be aligned with the sensor’s axes, and its origin is assumed to be located at the center of the accelerometer triad. The measurement model for the gyroscope angular velocity is modeled by

(6) $$y_{\omega }=\omega_{s}^{s}+e_{\omega }^{s},$$

where $y_{\omega }\in R^{3\times 1}$ is the measured angular velocity, and $e_{\omega }^{s}∼N\left(0,\Sigma_{\omega ,s}\right)$ is the measurement noise. Measured angular velocities can be expressed in terms of the body segment’s angular velocities, which can be expressed in terms of the system’s current generalized coordinates and velocities [33] as

(7) $$y_{\omega }=R^{sb}R^{bn}\left(q\right)\omega_{b}^{n}\left(q,\dot{q}\right)+e_{\omega }^{s}.$$

Here, two rotation matrices are introduced. First, $R^{sb}$ is the constant rotation matrix between the body frame *b* and the sensor frame *s*, which is assumed to be known. Second, $R^{bn}$ is the rotation matrix that describes the orientation transformation from the navigation frame *n* to the body segment frame *b* as a function of the joint angles. Note that the body frame and sensor frame have the same angular velocity, provided that the velocities are expressed in a common frame.

The IMU’s accelerometer measures the linear accelerations that the sensor experiences, including the acceleration due to gravity g. The measured accelerations depend on the current generalized joint angles, angular velocities and angular accelerations and can be expressed as

(8) $$y_{\dot{v}}=R^{sb}R^{bn}\left(q\right)\left(a_{s}^{n}\left(x\right)-g^{n}\right)+e_{\dot{v}}^{s}.$$

Here, $y_{\dot{v}}\in R^{3\times 1}$ is the measured linear acceleration, and $g^{n}\in R^{3\times 1}$ is the gravity vector expressed in the navigation frame. We model the accelerometer measurement noise as $e_{\dot{v}}^{s}∼N\left(0,\Sigma_{\dot{v}}\right)$. The acceleration $a_{s}^{n}\left(x\right)$ is defined in terms of the body’s angular velocity, angular acceleration and linear acceleration as

(9) $$a_{s}^{n}\left(x\right)=a_{b}^{n}\left(x\right)+\left[\alpha_{b}^{n}\left(x\right)\times \right]\left(R^{nb}\left(q\right)r_{bs}^{b}\right)+{\left[\omega_{b}^{n}\left(q,\dot{q}\right)\times \right]}^{2}\left(R^{nb}\left(q\right)r_{bs}^{b}\right).$$

**Figure 2:**
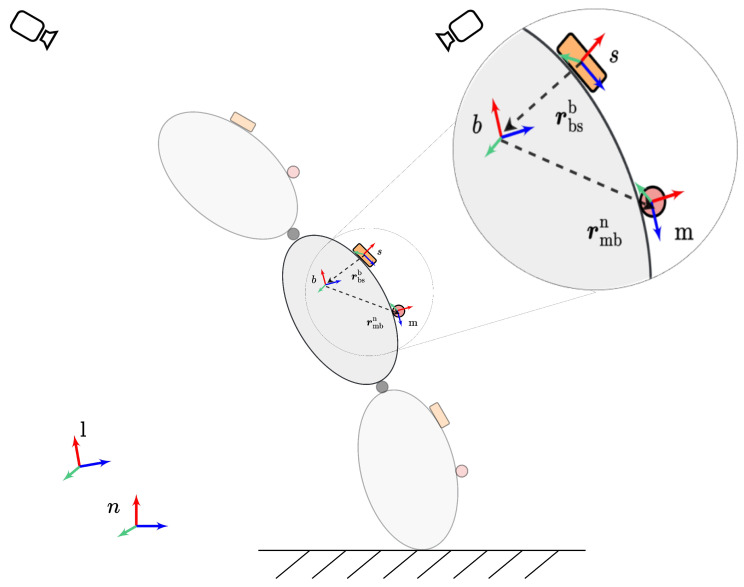
Definitions of the navigation frame *n*, the laboratory frame *l*, the body frame *b*, the IMU frame *s*, and the marker frame *m*. It also includes the translation vectors $r_{\mathrm{bs}}^{b}$ and $r_{\mathrm{mb}}^{n}$.

Here, $a_{b}^{n}$, $\alpha_{b}^{n}$ and $\omega_{b}^{n}$ are respectively the body segment’s linear acceleration, angular acceleration and angular velocity. These kinematic quantities are computed by propagating the joint angles, velocities, and accelerations, q, $\dot{q}$, and $\ddot{q}$, recursively through the multibody chain from the fixed base, where $\ddot{q}$ depends on τ through (2) and is evaluated by OpenSim. Furthermore, $r_{bs}^{b}$ is a translation vector expressed in the body frame *b* that locates the position of the IMU frame *s* to the body frame *b*, as shown in Figure 2. The rotation matrix $R^{\mathrm{nb}}\left(q\right)={\left(R^{\mathrm{bn}}\left(q\right)\right)}^{T}$ maps the body frame to the navigation frame.

### 2.3. Marker-Based Motion Capture and Zero-Torque Update Measurement Models

Marker-based motion-capture systems use multiple high-speed cameras fitted within a laboratory. After calibration, these systems provide marker position measurements with respect to a laboratory frame *l*. Each body segment can have any number of markers attached for a total number of markers $N_{M}$. We denote each individual marker by $m=1,\ldots ,N_{M}$. The marker position with respect to the lab frame can then be defined as

(10) $$y_{p}=p_{m}^{l}+e_{p}^{l},$$

where $y_{p}\in R^{3\times 1}$ is the marker position measured in the laboratory frame, and $e_{p}^{l}∼N\left(0,\Sigma_{p,m}\right)$ is the position measurement noise. Marker position measurements can be written in terms of the body position in the navigation frame as

(11) $$y_{p}=R^{\mathrm{lb}}\left(q\right)R^{\mathrm{bn}}\left(q\right)\left(p_{b}^{n}+r_{mb}^{n}\left(q\right)\right)+e_{p}^{l}.$$

The translation vector $r_{mb}^{n}\left(q\right)\in R^{3\times 1}$ is a vector that maps the position of the body segment frame *b* to the marker frame *m* expressed in the navigation frame *n*, as shown in Figure 2. The two matrices $R^{\mathrm{bn}}\left(q\right)\in R^{3\times 3}$ and $R^{\mathrm{lb}}\left(q\right)\in R^{3\times 3}$ map the navigation frame *n* to the body frame *b* and the body frame *b* to the laboratory *l*, respectively.

We also consider the occasional availability of zero-torque measurement updates. For this, virtual joint torque sensors $\phi =1,\ldots ,N_{D}$ that assume joint torque measurements of zero are expressed as

(12) $$y_{\tau }=0+e_{\tau }^{\phi },$$

where $y_{\tau }\in R^{1\times 1}$ is the joint zero-torque virtual measurement, and $e_{\tau }^{\phi }∼N\left(0,\Sigma_{\tau ,\phi }\right)$ is the virtual torque measurement noise.

### 2.4. Tightly Coupled Kinematics and Kinetics Estimation Algorithm

To couple the system dynamics with the measurement models (above), we employ an iterated extended Kalman filter (IEKF), specified as follows (see Algorithm 1). The multibody system dynamics are defined by the OpenSim model, which includes body segment masses, inertial properties, joint types, and sensor attachment locations. OpenSim provides the computational framework to evaluate the mass matrix $\bar{M}\left(q\right)$, velocity-dependent terms $C(q,\dot{q})$, and gravitational forces G(q) in the process model g(x) from (4). Additionally, OpenSim computes the kinematic quantities for the measurement model $h(x_{t})$ in (14), including rotation matrices $R^{bn}\left(q\right)$ in (7) and (8), body segment accelerations $a_{b}^{n}$, angular velocities $\omega_{b}^{n}$, and angular accelerations $\alpha_{b}^{n}$. Furthermore, OpenSim enables the computation of the process and measurement Jacobians required for the IEKF [23,35]. The dynamic model is discretized at the sensor measurement frequency (100 Hz) to predict the future state at time t+1 by numerically integrating the equations of motion using OpenSim’s Runge–Kutta integrator [35], resulting in a discrete-time update equation

(13) $$x_{t}=f\left(x_{t-1},\Delta t\right)+w_{t},$$

where $x_{t}$ denotes the state at time instance *t*, $f(x_{t},\Delta t)$ represents the numerical integration of the continuous dynamics in (4) over the time step Δt, and $w_{t}∼N\left(0,Q_{\Delta }\right)$ is the discrete-time process noise. The algorithm measurement model that includes all the previously discussed sensor measurement models can then be expressed as

(14) $$y_{t}=\left(\begin{array}{c} y_{\omega ,t,s_{1}}\\ y_{\dot{v},t,s_{1}}\\ y_{p,t,m_{1}}\\ y_{\tau ,t,\phi_{1}}\\ \vdots \\ y_{\omega ,t,s_{N_{I}}}\\ y_{\dot{v},t,s_{N_{I}}}\\ y_{p,t,m_{N_{M}}}\\ y_{\tau ,t,\phi_{N_{D}}} \end{array}\right)=\underset{h(x_{t})}{\underset{︸}{\left(\begin{array}{c} h_{\omega ,t,1}\left(x_{t}\right)\\ h_{\dot{v},t,1}\left(x_{t}\right)\\ h_{p,t,1}\left(x_{t}\right)\\ h_{\tau ,t,1}\left(x_{t}\right)\\ \vdots \\ h_{\omega ,t,N_{I}}\left(x_{t}\right)\\ h_{\dot{v},t,N_{I}}\left(x_{t}\right)\\ h_{p,t,N_{M}}\left(x_{t}\right)\\ h_{\tau ,t,N_{D}}\left(x_{t}\right) \end{array}\right)}}+e_{\mathrm{meas},t}.$$

Here, $h(x_{t})$ is the measurement model, $e_{\mathrm{meas},t}∼N\left(0,R\right)$ is the total measurement error, and $R\in R^{\left(6N_{I}+3N_{M}+N_{D}\right)\times \left(6N_{I}+3N_{M}+N_{D}\right)}$ is the measurement error covariance.
**Algorithm 1:** Tightly coupled motion-capture algorithm (TCMA)  **Input:** Sensor measurements $y_{t}$, an initial state estimate ${\hat{x}}_{0}$ with covariance ${\hat{P}}_{0}$, process and measurement noise covariance matrices Q and R; see (4), (13) and (14).  **Output:** Estimates of the system states ${\hat{x}}_{t}$ and the respective covariance ${\hat{P}}_{t}$ for t=1,…T.   1: **for** t=1,…,T **do**   2: **Time update**  Compute the state prediction by numerically integrating the noise-free dynamics in (4) from the previous estimate ${\hat{x}}_{t-1}$ over one time step Δt,(15)$$\begin{array}{cc} {\overset{ˇ}{x}}_{t} & =f({\hat{x}}_{t-1},\Delta t). \end{array}$$
      Compute the predicted state covariance as(16)$$\begin{array}{cc} {\overset{ˇ}{P}}_{t} & =F_{t-1}{\hat{P}}_{t-1}F_{t-1}^{T}+Q, \end{array}$$      where the discrete-time state transition Jacobian is approximated by discretizing the continuous-time Jacobian as $F_{t-1}\approx \exp\left(G_{t-1}\Delta t\right)$, with $G_{t-1}={\left.\frac{\partial g(x)}{\partial x}\right|}_{x={\hat{x}}_{t-1}}$ computed numerically.  3: **Measurement update**  4:   **for** k=1,…,ϵ **until** ${\bar{x}}_{t}^{k+1}\approx {\bar{x}}_{t}^{k}$ **do**  5:    Update the state with measurements $y_{t}$ as(17)$$\begin{array}{cc} {\bar{x}}_{t}^{k+1} & ={\overset{ˇ}{x}}_{t}+K_{t}^{k}\left(y_{t}-h_{t}\left({\bar{x}}_{t}^{k}\right)-H_{t}^{k}\left({\overset{ˇ}{x}}_{t}-{\bar{x}}_{t}^{k}\right)\right), \end{array}$$
       where $K_{t}^{k}={\overset{ˇ}{P}}_{t}{\left(H_{t}^{k}\right)}^{T}{\left(H_{t}^{k}{\overset{ˇ}{P}}_{t}{\left(H_{t}^{k}\right)}^{T}+R\right)}^{-1}\mathrm{and}\;$
$H_{t}^{k}={\left.\frac{\partial h_{t}\left(x_{t}\right)}{\partial x_{t}}\right|}_{x_{t}={\bar{x}}_{t}^{k}}$.  6:   Compute the filtered state and covariance(18)$$\begin{array}{c} {\hat{x}}_{t}={\bar{x}}_{t}^{k+1},\;{\hat{P}}_{t}=\left(I-K_{t}^{k}H_{t}^{k}\right){\overset{ˇ}{P}}_{t}. \end{array}$$

The process of estimating the system states ${\hat{x}}_{t}$ and their covariance ${\hat{P}}_{t}$ using an IEKF is described in Algorithm 1. The first step is prediction, in which the state and covariance are predicted as ${\overset{ˇ}{x}}_{t}$ and ${\overset{ˇ}{P}}_{t}$ respectively. In this step the Jacobian $G=\frac{\partial g(x)}{\partial x}\in R^{3N_{D}\times 3N_{D}}$ is calculated to compute ${\overset{ˇ}{P}}_{t}$. In the second step the algorithm uses the measurements to iteratively update the values of the state and covariance. In this step the Jacobian $H=\frac{\partial h(x_{t})}{\partial x_{t}}\in R^{\left(6N_{I}+3N_{M}+N_{D}\right)\times 3N_{D}}$ is calculated to compute the Kalman gain $K_{t}$, the updated states ${\hat{x}}_{t}$ and updated state covariance ${\hat{P}}_{t}$. To handle the nonlinearities of the measurement model, the IEKF re-linearizes around the most recent state estimate at each iteration within the same time step, refining the state estimate until convergence, as shown in Algorithm 1. This reduces the linearization error compared to the standard EKF, which linearizes only once around the predicted state. The discrete-time state transition Jacobian $F_{t-1}$ is approximated via the matrix exponential of the continuous-time Jacobian rather than by perturbing the numerical integrator directly, as the latter would require $3N_{D}$ additional integrations per time step with no observable gain in accuracy.

### 2.5. Real-World Testing on Physical Systems

**Figure 3:**
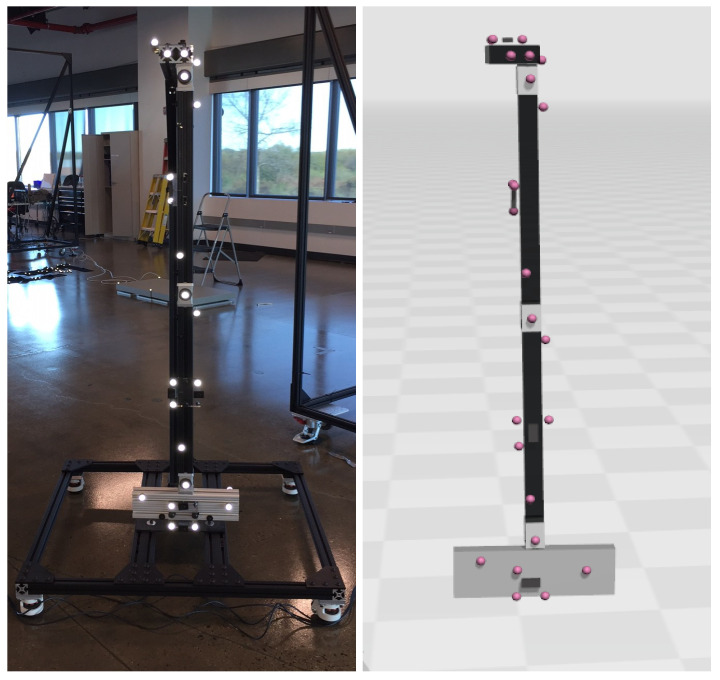
Experimental setup of a four-segment 3-DoF pendulum setup from Johns Hopkins Applied Physics Laboratory (**left**), where optical markers and an IMU are attached to each body segment. On the **right** is an OpenSim model with corresponding IMU and marker placements.

To validate our approach, we performed two separate sets of experiments on two different systems. In the first setup we used a 3-degree-of-freedom (DoF) physical four-link pendulum consisting of aluminum segments connected by roller-bearing hinge joints, as shown in Figure 3. Each body segment was equipped with an APDM Opal inertial sensor (APDM Wearable Technologies, Portland, OR, USA) and a set of optical motion-capture markers tracked by a Vicon motion-capture system (Vicon Motion Systems Ltd., Oxford, UK). Experiments were conducted on this passive multibody system, in which the lowest body segment, shown in silver in Figure 3, was raised to a certain height and then released to swing under its own weight; the applied joint torques are assumed to be zero upon release. We evaluated Algorithm 1 (TCMA) using three different combinations of sensor measurements to estimate the pendulum motion:

- IMU measurements only (${\mathrm{TCMA}}_{I}$);
- IMU and marker measurements (${\mathrm{TCMA}}_{I\&M}$);
- IMU, marker, and virtual zero-torque measurements (${\mathrm{TCMA}}_{I,M\&0T}$).

We compared the output of our algorithm with these different measurement combinations against OpenSim marker-based IK/ID (${\mathrm{IK}}_{M}$/${\mathrm{ID}}_{M}$) and OpenSense IMU orientation-based IK/ID (${\mathrm{IK}}_{\mathrm{Io}}$/${\mathrm{ID}}_{\mathrm{Io}}$) across six trials, each with different initial generalized joint angles. While marker-based IK/ID is commonly used as a reference standard, we additionally include ${\mathrm{TCMA}}_{I\&M}$ as a reference. This allows a comparison of OpenSense (which uses only IMU orientation estimates) against a method that integrates both measurement modalities.

**Figure 4:**
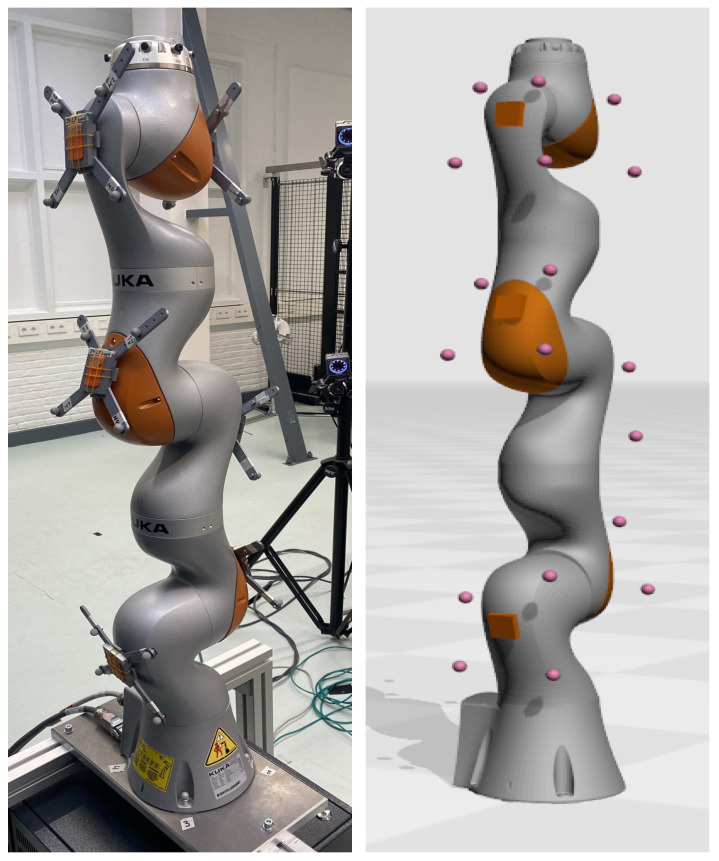
The experimental setup for the robot experiment using the KUKA LBR 7 iiwa R800 collaborative robot, where a set of 4 markers and an IMU were placed on each body segment (**left**). On the **right**, the OpenSim model that describes the system is shown.

The second experimental setup was a KUKA LBR 7 iiwa R800 robot (Figure 4) consisting of 7 bodies and 6 revolute joints. Markers tracked by an OptiTrack motion-capture system (NaturalPoint, Inc., Corvallis, OR, USA) and Xsens MTw2 IMUs (Movella/Xsens, Enschede, The Netherlands) were fitted on all robot body segments except the base and the end-effector. Five separate trials were conducted in which we extracted the markers’ 3D positions, IMU accelerations and angular velocities, as well as ground-truth joint angles and torques from the robot’s internal encoders and sensors. We applied the TCMA to estimate the robot motion using two measurement configurations: IMU-only measurements in 4 trials and combined IMU and marker-based measurements in 1 trial. For the combined measurement trial, we also compared results with ${\mathrm{IK}}_{M}$/${\mathrm{ID}}_{M}$. The OpenSense ${\mathrm{IK}}_{\mathrm{Io}}$/${\mathrm{ID}}_{\mathrm{Io}}$ method was not feasible due to magnetic disturbances from the robot’s metallic structure and motors, which compromise magnetometer-based orientation estimates [15]. For the KUKA robot setup, all IMUs were pre-calibrated before each trial to remove accelerometer and gyroscope biases.

The measurement noise covariance matrix R was constructed using the KUKA robot setup by computing the sample covariance of residuals between sensor measurements and the corresponding encoder-derived robot states, treating the encoder measurements as ground truth given their high accuracy [36]. The process noise covariance $Q_{\tau }$ was set as a diagonal matrix, with elements manually tuned to approximately 1 (Nm)^2^ to balance dynamic flexibility and estimation accuracy. These same R and $Q_{\tau }$ values were used for both the KUKA and pendulum experiments without further adjustment, as the algorithm exhibited low sensitivity to the precise values of R, and the chosen $Q_{\tau }$ provided adequate performance across both experimental setups.

## 3. Results

We present the motion estimated by the TCMA using the sensor measurement combinations described in Section 2.5. For each experimental setup, we report root-mean-square difference (RMSD) values across all trials and illustrate the estimated joint angles and torques for a representative trial.

**Figure 5:**
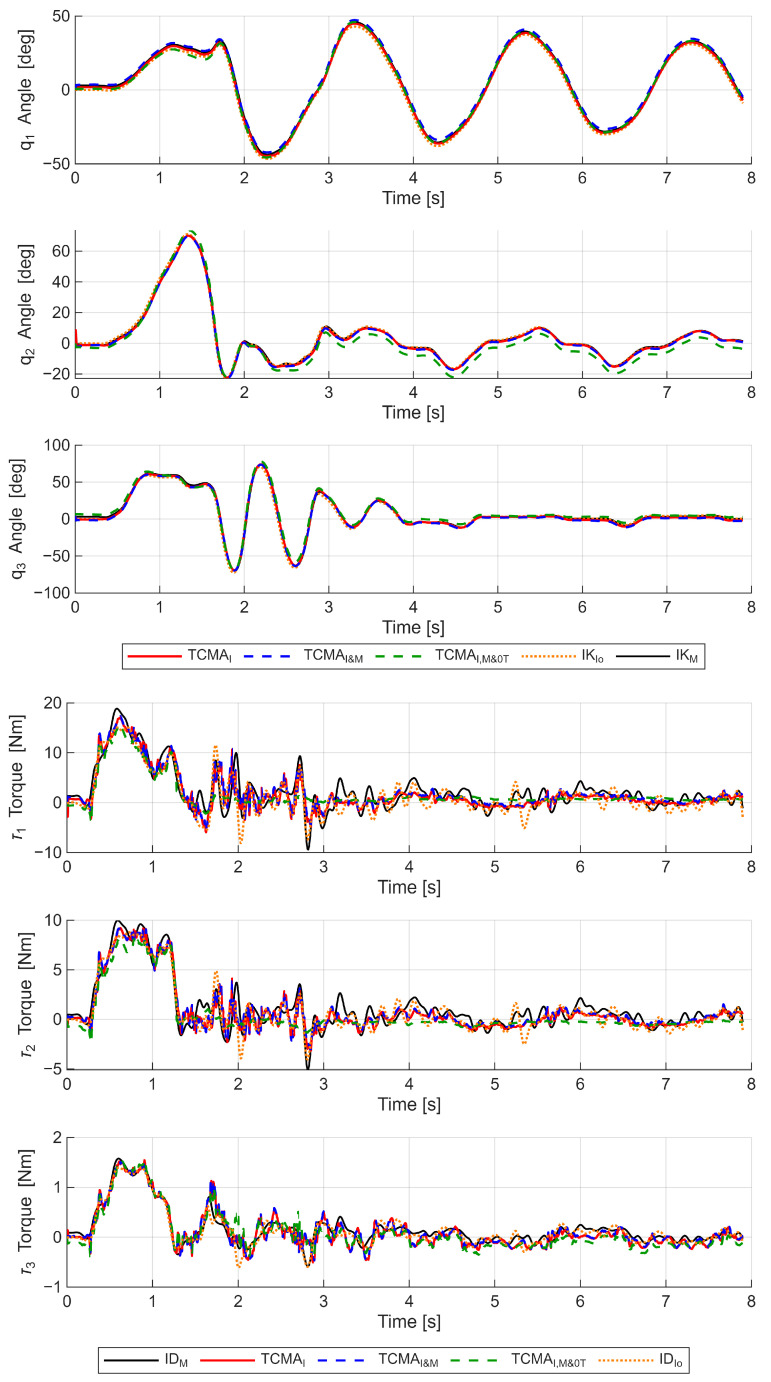
Estimated joint angles (**top**) and torques (**bottom**) for a randomly selected pendulum trial (trial 3) using ${\mathrm{TCMA}}_{I}$, ${\mathrm{TCMA}}_{I\&M}$, and ${\mathrm{TCMA}}_{I,M\&0T}$, plotted against ${\mathrm{IK}}_{\mathrm{Io}}$/${\mathrm{ID}}_{\mathrm{Io}}$ and ${\mathrm{IK}}_{M}$/${\mathrm{ID}}_{M}$.

Figure 5 shows representative results from a randomly selected pendulum trial (trial 3). At time 0 s, the pendulum was in its initial position, as shown in Figure 3. The pendulum was raised and then released to swing freely under its own weight from 1.2 s to 7.8 s. All other trials followed the same procedure with different initial joint angles. The virtual zero-torque measurements shown in Figure 5 were only applied to ${\mathrm{TCMA}}_{I,M\&0T}$ after the pendulum was released. Table 1 presents RMSD values across all six pendulum trials, comparing estimates from the TCMA using the different measurement combinations (${\mathrm{TCMA}}_{I}$, ${\mathrm{TCMA}}_{I\&M}$, and ${\mathrm{TCMA}}_{I,M\&0T}$), alongside ${\mathrm{IK}}_{\mathrm{Io}}$/${\mathrm{ID}}_{\mathrm{Io}}$, evaluated against ${\mathrm{IK}}_{M}$/${\mathrm{ID}}_{M}$ and ${\mathrm{TCMA}}_{I\&M}$ as reference standards. Estimates from the TCMA showed small deviations in joint angles (≤4.23°) and joint torques (≤3.02 Nm) when compared to ${\mathrm{IK}}_{M}$/${\mathrm{ID}}_{M}$. However, a steady offset of approximately 2° is observed in the $q_{2}$ estimate from ${\mathrm{TCMA}}_{I,M\&0T}$ after 2.4 s compared with the other $q_{2}$ estimates (Figure 5).

**Figure 6:**
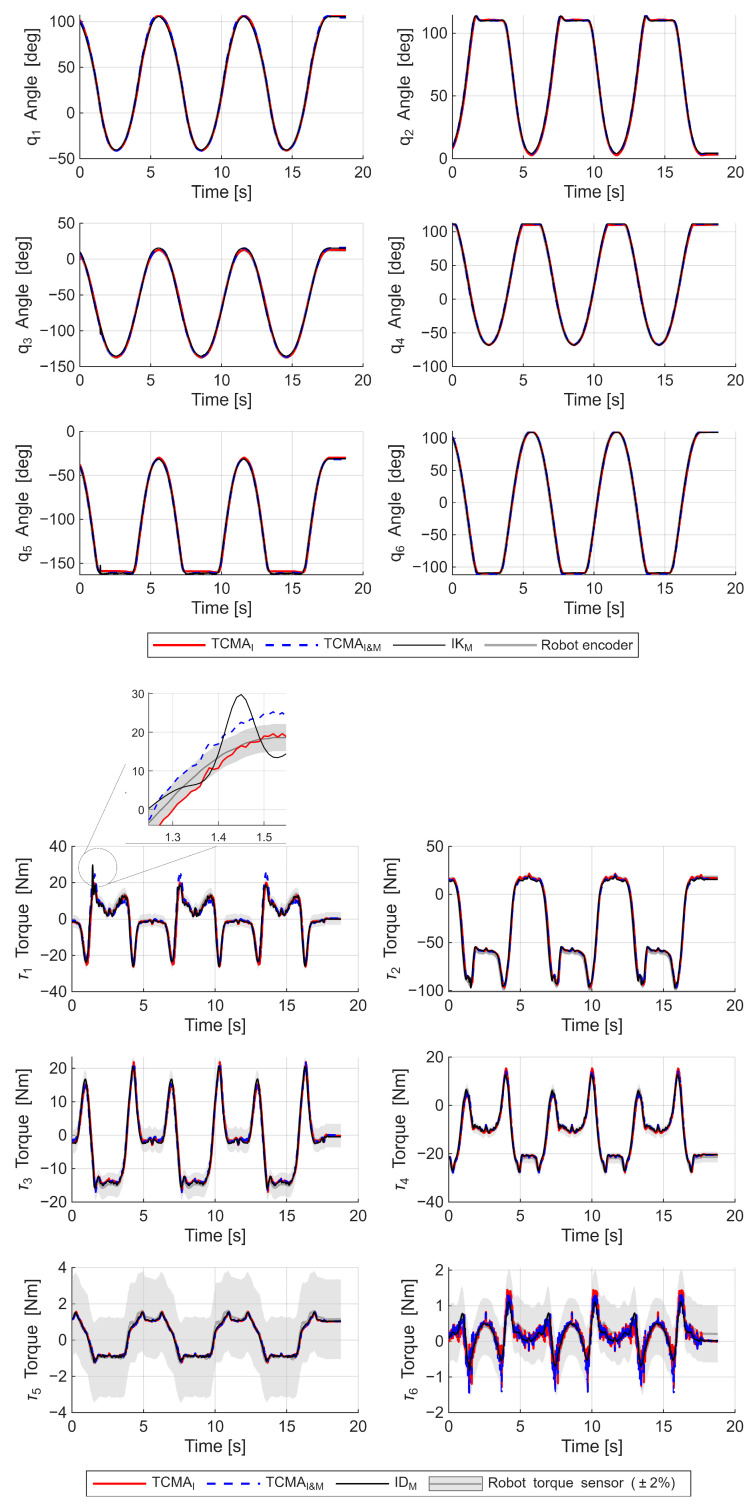
Estimated joint angles (**top**) and torques (**bottom**) for trial 1 using ${\mathrm{TCMA}}_{I}$ and ${\mathrm{TCMA}}_{I\&M}$, plotted against ${\mathrm{IK}}_{M}$/${\mathrm{ID}}_{M}$ and the robot measurements. The robot torque is shown as the moving-average mean with a shaded ±2% uncertainty band for the maximum rated joint torque.

**Figure 7:**
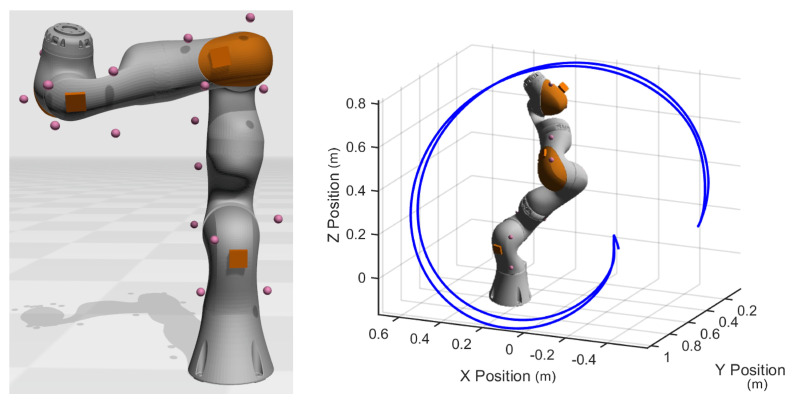
Robot trial 1: (**left**) initial pose and (**right**) end-effector motion path.

The KUKA robot experiments show similar performance. Joint angle and torque estimates from the TCMA, using either IMU-only measurements (${\mathrm{TCMA}}_{I}$) or combined IMU and marker measurements (${\mathrm{TCMA}}_{I\&M}$), are compared with ${\mathrm{IK}}_{M}$/${\mathrm{ID}}_{M}$ and the robot’s internal encoders and torque sensors (Figure 6). The robot’s initial pose is shown in Figure 7. The shaded regions in Figure 6 indicate the robot torque sensor accuracy (±2% of maximum torque, ranging from 176 Nm for joint 1 to 40 Nm for joint 6 [36,37]), providing context for evaluating whether our estimates fall within sensor uncertainty. The robot torque measurements shown are smoothed in Figure 6 using a 20-sample moving average filter to reduce high-frequency noise.

In the trial shown in Figure 6, all joints except the end-effector were simultaneously actuated. This was consistent across all five trials. Figure 7 shows the resulting end-effector motion path for trial 1. For trial 1, ${\mathrm{TCMA}}_{I}$ showed a maximum joint angle error of 3.24°, while ${\mathrm{TCMA}}_{I\&M}$ showed a maximum joint angle error of 2.84°. Torque errors were below 4.27 Nm for ${\mathrm{TCMA}}_{I}$ and 3.71 Nm for ${\mathrm{TCMA}}_{I\&M}$ (Table 2). Similarly, across trials 2 to 5, ${\mathrm{TCMA}}_{I}$ maintained maximum errors of 3.01° for joint angles and 2.33 Nm for joint torques (Table 3). Processing the experimental data with the TCMA took approximately 21.5 times longer than the data duration for the robot experiments (e.g., 412 s for a 19.2 s trial) and 9.8 times longer for the pendulum experiments (e.g., 77 s for a 7.9 s trial). Computations were performed using MATLAB R2025b on a Dell Latitude 7440 laptop equipped with an Intel Core i7-1365U processor (13th Gen, 1.80 GHz) with 16 GB RAM.

## 4. Discussion

We developed a tightly coupled motion-capture algorithm (TCMA) that is based on an iterated extended Kalman filter (IEKF) to tightly couple IMU measurements and a multibody system model to estimate the system kinematics and kinetics directly. Unlike traditional IMU-based methods such as OpenSense [22], our formulation does not depend on computing sensor orientations. Hence, it can be used in indoor applications, where there are often strong ferromagnetic disturbances affecting the magnetometer, as demonstrated in the robot experiment.

The results of the trials conducted on the pendulum experimental setup indicate that, with respect to ${\mathrm{IK}}_{M}$, the ${\mathrm{TCMA}}_{I}$ joint angle RMSD values were on average 19% lower than those from ${\mathrm{IK}}_{\mathrm{Io}}$ (Table 1). The maximum RMSD between the ${\mathrm{TCMA}}_{I}$ joint angle estimates and ${\mathrm{IK}}_{M}$ was 3.75°, compared to 6.56° for ${\mathrm{IK}}_{\mathrm{Io}}$ (Table 1).

Across all six pendulum trials, torque estimates from the TCMA with different measurement combinations were within ∼3 Nm RMSD of the marker-based ID (Table 1). The TCMA with different measurement combinations, particularly the virtual zero-torque measurements, produced torque estimates that fluctuated around zero from about 3 s onward (Figure 5, Table 1). This behavior aligns with the expected physical dynamics of the pendulum, where only the low friction of the joints is present during free passive swinging. However, the lack of joint friction in the model may have caused the observed offset in ${\mathrm{TCMA}}_{I,M\&0T}$’s $q_{2}$ estimate (Figure 5). Since the virtual zero-torque measurements pull the estimated torques toward zero, any unmodeled friction torque cannot be absorbed by the torque state and instead shows up as a small bias in the joint angles. This effect is most visible at $q_{2}$, which is the only intermediate joint and therefore the only one coupling two freely swinging segments, so friction contributions from motion on both sides affect it.

The robot trials further demonstrate the ability of our approach to provide accurate motion estimates in environments with strong magnetic distortions, where using the OpenSense approach was not possible. Five motion paths were selected to exploit the high maneuverability of the robot (Figure 7), showcasing the capability of our method to track the corresponding kinematics and kinetics (Figure 6). The ${\mathrm{TCMA}}_{I}$ joint angle estimates were consistent with measurements in the pendulum trials, demonstrating joint angle RMSD values below 3.25° with respect to the joint encoders (Table 2 and Table 3).

The torque estimates produced by ${\mathrm{TCMA}}_{I}$ were within 4.27 Nm RMSD of the robot’s integrated torque sensor measurements across all trials (Table 2 and Table 3). Furthermore, the TCMA tracked abrupt changes in the torque profile. For example, around 1.4 s in the $\tau_{1}$ estimate, the motion capture ID overshot by approximately 12 Nm, whereas ${\mathrm{TCMA}}_{I}$ did not (Figure 6). In the worst-case scenario, the RMSD of ${\mathrm{TCMA}}_{I}$ exceeded the torque sensors’ uncertainty by 0.5% in the estimate of $\tau_{2}$ in trial 1, where the torque RMSD was 4.27 Nm (Table 2).

A key advantage of our approach is the elimination of kinematic filtering requirements inherent to traditional ID methods. Conventional ID approaches (both OpenSense ID and marker-based ID) require low-pass filtering of kinematic data to reduce high-frequency artifacts introduced by inverse kinematics. Hence, selecting an appropriate cutoff frequency presents a significant challenge, as different body segments may move at varying frequencies, making a single cutoff frequency suboptimal across all joints [38]. This issue is particularly relevant in motions involving contact events, where making and breaking contact with the environment introduces higher-frequency dynamics, while limb movements remain predominantly low-frequency [39]. Our algorithm mitigates this limitation by directly estimating kinetics from the dynamic model without requiring explicit cutoff frequency selection, thereby reducing sensitivity to segment-dependent motion speeds and arbitrary filtering choices. This was evident in both the pendulum experiment, where torque estimates naturally fluctuated around zero during free swinging (Figure 5), and the robot experiment, where abrupt torque changes were accurately tracked (Figure 6).

The inclusion of markers in the TCMA measurement model improved accuracy, with a 15–40% reduction versus the robot joint angle encoder RMSD (Table 2). Additionally, the virtual zero-torque measurements applied during the pendulum experiment produced the smoothest torque profile (Figure 5). These results show the effectiveness of combining different measurement models to enhance accuracy. For example, IMU measurements can be combined with pressure insoles or other force reaction measurements to capture human kinematics and kinetics outside the laboratory.

While our results from multibody mechanical systems are very encouraging and highlight distinct advantages, they rely on several assumptions that may limit the broader applicability of our approach. First, the locations of the IMUs were assumed to be rigid (i.e., free of soft tissue artifacts) and accurately known a priori, which is generally not the case in practice. In this work, we used motion-capture measurements to calibrate the sensor-to-body locations during our experiments. Second, although the TCMA exhibited low sensitivity to the measurement noise covariance matrix R, it was more sensitive to the torque process noise $Q_{\tau }$. However, the development of adaptive process noise estimation methods lies beyond the scope of this work. Although the TCMA is sequential and thus potentially real-time-capable, currently, it does not yet meet real-time computational requirements.

Future improvements could address the sensor-to-body calibration limitation by incorporating an automatic sensor registration algorithm, which would solve for the sensor orientation and translation with respect to the body segment using the model’s joint properties in addition to the sensor measurements [40]. The algorithm could also include an adaptive process and measurement covariance estimator to improve estimation accuracy, particularly when applied to different multibody systems in different environments. Additionally, estimating the gyroscope bias within the system states could further enhance accuracy. The TCMA also facilitates integration of additional sensor modalities, such as markerless motion-capture systems and force sensors, by defining corresponding measurement models in terms of the system states. This could enable estimation of additional quantities, such as joint contact forces and loading, while also improving overall motion estimation accuracy. An automatically differentiable model would provide exact Jacobians instead of numerical perturbations, potentially improving both TCMA accuracy and computational efficiency. Since TCMA uses the dynamics in OpenSim, the same approach can be applied to other models, including human musculoskeletal models that use joint and coordinate definitions commonly used by movement scientists and clinicians. This compatibility with existing biomechanical frameworks could facilitate broader adoption of IMU-based motion capture in clinical settings.

## 5. Conclusions

We presented a tightly coupled (IEKF-based) motion-capture algorithm (TCMA) that integrates the system’s dynamic model with different combinations of sensor measurements (primarily IMU accelerometer and gyroscope measurements) to directly estimate both the kinematics and kinetics of a multibody system. Comparing our IMU TCMA estimates against the ground truth from optical motion capture and robot sensors, we demonstrate an accuracy of less than 3.8° compared to marker-based optical motion capture, which is prevalent in human biomechanical studies.

Integrating multibody dynamics and related constraints into the estimation process ensures that the estimated states are inherently consistent with both kinematic and kinetic relationships, thereby mitigating the inconsistencies typically observed in two-step approaches. Our TCMA enabled reliable performance in magnetically disturbed environments, as demonstrated via the robot experiment, where magnetometer-dependent methods such as OpenSense were not feasible. Furthermore, unlike traditional inverse dynamics methods that require low-pass filtering with arbitrary cutoff frequencies, our algorithm provides direct kinetic estimates from the dynamic model. Our algorithm also facilitates integration of additional sensing modalities such as markerless motion-capture systems, enabling further enhancement of estimation accuracy. These results are promising for deploying IMU-based motion capture in real-world environments where optical systems are impractical.

## Figures and Tables

**Table 1:** Joint angle RMSD (°) and joint torque RMSD (Nm) across six pendulum trials, with ${\mathrm{IK}}_{M}$/${\mathrm{ID}}_{M}$ and ${\mathrm{TCMA}}_{I\&M}$ as references respectively (${\mathrm{RMSD}}_{\left({\mathrm{IK}}_{M}/{\mathrm{ID}}_{M}\right)}$|${\mathrm{TCMA}}_{I\&M}$). Bold values indicate the highest joint angle and joint torque RMSD values per trial across all methods, reported separately for the two RMSD references.

Trial	Metric	RMSD vs. ${\mathrm{IK}}_{M}$/${\mathrm{ID}}_{M}$ | RMSD vs. ${\mathrm{TCMA}}_{I\&M}$				
		${\mathrm{TCMA}}_{I}$	${\mathrm{TCMA}}_{I\&M}$	${\mathrm{TCMA}}_{I,M\&0T}$	${\mathrm{IK}}_{\mathrm{Io}}$/${\mathrm{ID}}_{\mathrm{Io}}$	${\mathrm{IK}}_{M}$/${\mathrm{ID}}_{M}$
1	$q_{1}$ (°)	1.27 | 0.10	1.28 | –	1.29 | 0.10	0.92 | 2.13	– | 1.28
	$q_{2}$ (°)	0.91 | 0.12	0.93 | –	0.99 | 0.09	0.92 | 1.28	– | 0.93
	$q_{3}$ (°)	3.49 | 0.12	3.54 | –	4.23 | 0.93	**6.56** | 3.32	– | **3.54**
	$\tau_{1}$ (Nm)	1.05 | 0.03	**1.07** | –	0.92 | 0.47	0.78 | **1.56**	– | 1.07
	$\tau_{2}$ (Nm)	0.39 | 0.01	0.40 | –	0.34 | 0.28	0.39 | 0.66	– | 0.40
	$\tau_{3}$ (Nm)	0.07 | 0.00	0.07 | –	0.11 | 0.07	0.15 | 0.16	– | 0.07
2	$q_{1}$ (°)	1.10 | 0.11	1.11 | –	1.11 | 0.10	1.54 | 2.26	– | 1.11
	$q_{2}$ (°)	0.94 | 0.17	0.97 | –	1.02 | 0.11	2.21 | 2.60	– | 0.97
	$q_{3}$ (°)	3.75 | 0.10	3.72 | –	4.16 | 0.75	**5.50** | **4.05**	– | 3.72
	$\tau_{1}$ (Nm)	**1.70** | 0.05	**1.70** | –	1.33 | 0.82	**1.70** | **1.91**	– | 1.70
	$\tau_{2}$ (Nm)	1.01 | 0.01	1.00 | –	0.79 | 0.48	0.75 | 1.09	– | 1.00
	$\tau_{3}$ (Nm)	0.18 | 0.00	0.18 | –	0.21 | 0.12	0.12 | 0.26	– | 0.18
3	$q_{1}$ (°)	1.34 | 0.10	1.35 | –	1.38 | 0.10	1.00 | 1.67	– | 1.35
	$q_{2}$ (°)	0.76 | 0.16	0.79 | –	0.87 | 0.13	1.10 | 1.27	– | 0.79
	$q_{3}$ (°)	1.64 | 0.09	1.66 | –	1.86 | 0.40	**3.18** | **2.81**	– | 1.66
	$\tau_{1}$ (Nm)	1.23 | 0.05	1.24 | –	1.25 | 0.66	**1.45** | **1.52**	– | 1.24
	$\tau_{2}$ (Nm)	0.63 | 0.01	0.63 | –	0.62 | 0.39	0.64 | 0.71	– | 0.63
	$\tau_{3}$ (Nm)	0.14 | 0.00	0.14 | –	0.18 | 0.09	0.06 | 0.16	– | 0.14
4	$q_{1}$ (°)	1.28 | 0.13	1.27 | –	1.29 | 0.11	1.38 | 2.57	– | 1.27
	$q_{2}$ (°)	1.19 | 0.38	1.11 | –	1.22 | 0.13	0.65 | 1.07	– | 1.11
	$q_{3}$ (°)	2.75 | 0.28	2.69 | –	3.08 | 0.56	**3.38** | **3.91**	– | 2.69
	$\tau_{1}$ (Nm)	**1.53** | 0.18	1.52 | –	1.42 | 0.71	1.34 | **2.01**	– | 1.52
	$\tau_{2}$ (Nm)	0.76 | 0.05	0.76 | –	0.69 | 0.43	0.60 | 0.89	– | 0.76
	$\tau_{3}$ (Nm)	0.14 | 0.00	0.14 | –	0.18 | 0.10	0.08 | 0.18	– | 0.14
5	$q_{1}$ (°)	1.05 | 0.12	1.04 | –	1.05 | 0.11	0.86 | 1.51	– | 1.04
	$q_{2}$ (°)	1.24 | 0.58	1.18 | –	1.18 | 0.18	2.09 | 2.29	– | 1.18
	$q_{3}$ (°)	2.67 | 0.68	2.65 | –	**2.94** | 0.56	2.37 | 2.60	– | **2.65**
	$\tau_{1}$ (Nm)	**1.85** | 0.07	1.85 | –	1.74 | 0.69	1.18 | **2.06**	– | 1.85
	$\tau_{2}$ (Nm)	1.02 | 0.07	1.02 | –	0.95 | 0.41	0.60 | 1.10	– | 1.02
	$\tau_{3}$ (Nm)	0.26 | 0.02	0.26 | –	0.26 | 0.09	0.05 | 0.26	– | 0.26
6	$q_{1}$ (°)	1.45 | 0.80	1.27 | –	1.29 | 0.19	1.09 | 1.48	– | 1.27
	$q_{2}$ (°)	1.75 | 0.43	1.64 | –	1.67 | 0.24	1.43 | 1.51	– | 1.64
	$q_{3}$ (°)	**3.72** | 1.64	3.08 | –	3.04 | 1.11	3.23 | 2.80	– | **3.08**
	$\tau_{1}$ (Nm)	**3.02** | 0.52	2.92 | –	2.51 | 0.84	1.40 | 2.85	– | **2.92**
	$\tau_{2}$ (Nm)	1.72 | 0.22	1.68 | –	1.47 | 0.50	0.64 | 1.59	– | 1.68
	$\tau_{3}$ (Nm)	0.48 | 0.03	0.48 | –	0.47 | 0.10	0.09 | 0.49	– | 0.48

**Table 2:** Joint angle and torque RMSDs for robot trial 1, comparing ${\mathrm{TCMA}}_{I}$, ${\mathrm{TCMA}}_{I\&M}$, and ${\mathrm{IK}}_{M}$/${\mathrm{ID}}_{M}$ against the robot measurements. Bold values indicate the highest joint angle and joint torque RMSD values across all methods, with the robot measurements as the reference.

Joint	Angle RMSD (°) | Torque RMSD (Nm)		
	${\mathrm{TCMA}}_{I}$	${\mathrm{TCMA}}_{I\&M}$	${\mathrm{IK}}_{M}$/${\mathrm{ID}}_{M}$
$q_{1}$ | $\tau_{1}$	1.76 | 1.99	1.37 | 2.23	0.33 | 1.67
$q_{2}$ | $\tau_{2}$	1.54 | **4.27**	0.84 | **3.71**	0.66 | **2.97**
$q_{3}$ | $\tau_{3}$	1.44 | 1.41	1.45 | 1.13	**1.16** | 1.01
$q_{4}$ | $\tau_{4}$	2.31 | 1.64	1.45 | 1.51	0.75 | 1.25
$q_{5}$ | $\tau_{5}$	2.20 | 0.35	1.22 | 0.34	0.48 | 0.33
$q_{6}$ | $\tau_{6}$	**3.24** | 0.37	**2.84** | 0.38	**1.16** | 0.33

**Table 3:** Joint angle and torque RMSDs for robot trials 2–5 for ${\mathrm{TCMA}}_{I}$ versus the robot measurements. Bold values indicate the highest joint angle and joint torque RMSD values per trial, with the robot measurements as the reference.

Joint	Angle RMSD (°) | Torque RMSD (Nm)			
	Trial 2	Trial 3	Trial 4	Trial 5
$q_{1}$ | $\tau_{1}$	2.72 | 0.66	0.99 | 0.64	1.23 | 0.46	1.58 | 0.46
$q_{2}$ | $\tau_{2}$	1.86 | **2.33**	0.39 | **1.38**	0.87 | **1.65**	0.43 | **1.81**
$q_{3}$ | $\tau_{3}$	**3.01** | 0.82	**2.00** | 0.46	**2.12** | 0.33	**2.24** | 0.34
$q_{4}$ | $\tau_{4}$	2.06 | 0.45	0.77 | 0.32	1.24 | 0.36	0.95 | 0.38
$q_{5}$ | $\tau_{5}$	1.52 | 0.08	1.57 | 0.08	0.86 | 0.07	1.00 | 0.07
$q_{6}$ | $\tau_{6}$	1.73 | 0.23	1.42 | 0.23	1.77 | 0.22	1.26 | 0.23

## Data Availability

The TCMA is publicly available at https://github.com/Computational BiomechanicsLab/Tightly-Coupled-Motion-capture-Algorithm-TCMA (accessed on 1 May 2026).
